# Functional Grading of a Transversely Isotropic Hyperelastic Model with Applications in Modeling Tricuspid and Mitral Valve Transition Regions

**DOI:** 10.3390/ijms21186503

**Published:** 2020-09-05

**Authors:** Rajarshi Roy, Eric Warren, Yaoyao Xu, Caleb Yow, Rama S. Madhurapantula, Joseph P. R. O. Orgel, Kevin Lister

**Affiliations:** 1 Corvid Technologies, Mooresville, NC 28117, USA; yaoyao.xu@corvidtec.com (Y.X.); caleb.yow@corvidtec.com (C.Y.); kevin.lister@corvidtec.com (K.L.); 2 School of Medicine, Duke University, Durham, NC 27708, USA; eric.warren@duke.edu; 3 Department of Biology, Illinois Institute of Technology, Chicago, IL 60616, USA; rmadhura@iit.edu (R.S.M.); orgel@iit.edu (J.P.R.O.O.); 4 Pritzker Institute of Biomedical Science and Engineering, Illinois Institute of Technology, Chicago, IL 60616, USA; 5 Department of Biomedical Engineering, Illinois Institute of Technology, Chicago, IL 60616, USA

**Keywords:** constitutive modeling, X-ray diffraction, anisotropy, functionally graded material model, cardiac mechanics, multiscale modeling

## Abstract

Surgical simulators and injury-prediction human models require a combination of representative tissue geometry and accurate tissue material properties to predict realistic tool–tissue interaction forces and injury mechanisms, respectively. While biological tissues have been individually characterized, the transition regions between tissues have received limited research attention, potentially resulting in inaccuracies within simulations. In this work, an approach to characterize the transition regions in transversely isotropic (TI) soft tissues using functionally graded material (FGM) modeling is presented. The effect of nonlinearities and multi-regime nature of the TI model on the functional grading process is discussed. The proposed approach has been implemented to characterize the transition regions in the leaflet (LL), chordae tendinae (CT) and the papillary muscle (PM) of porcine tricuspid valve (TV) and mitral valve (MV). The FGM model is informed using high resolution morphological measurements of the collagen fiber orientation and tissue composition in the transition regions, and deformation characteristics predicted by the FGM model are numerically validated to experimental data using X-ray diffraction imaging. The results indicate feasibility of using the FGM approach in modeling soft-tissue transitions and has implications in improving physical representation of tissue deformation throughout the body using a scalable version of the proposed approach.

## 1. Introduction

### 1.1. Background and Motivation

Recent technological advancements in three-dimensional imaging, computational power and virtual reality have made it possible for digital human models to represent the human body with enhanced biofidelity and realism. For instance, surgical simulators have shown great promise in supplementing traditional training and planning of minimally invasive surgical procedures by recreating the surgical scene, with applications ranging from neurological to cardiovascular interventions [1]. Additionally, several whole-body finite element (FE) models [2,3] have been used to supplement post-mortem human subject impact data from mechanical insults such as blunt, penetrating and blast events to advance the fields of crashworthiness and impact biomechanics research. Both surgical simulators and whole-body injury-prediction models require detailed geometrical and biomechanical characterization of soft tissue to predict accurate tool-tissue interaction forces and damage propagation in the human body respectively. However, the vast body of literature on soft-tissue characterization focus on organ-specific homogeneous tissue properties [1,3], with lesser emphasis on the transition regions between various tissue types.

The study of morphological and biomechanical characteristics of tissue transition regions gains prominence in light of increasing evidence associating these regions with injuries resulting from multi-axial loading. For instance, the muscle–tendon junction (MTJ) has been hypothesized to be the site of sports overuse injuries such as calf-muscle tear and delayed onset muscle soreness (DOMS) [4]. In the case of cardiac tissue, the pathology of mitral valve insufficiency has been localized to the rupture of the chordae tendinae (CT) just below the insertion to the heart wall leaflet (LL) [5]. These observations have spurred researchers into investigating the damage mechanisms and underlying physiological bases related to the microstructure and the biomechanical signatures in the transition regions. Additionally, morphological and biomechanical models of these regions serve as useful design tools in the fabrication of biomimetic materials such as osteoimplants [6] and engineered tissue scaffolds [7].

Load bearing biological tissue are often uniquely characterized by multiscale hierarchical components displaying a continuous change from one composition or structure into another at their transition regions. For instance, the MTJ in the musculoskeletal system represents a transition between two hierarchical tissue types—at the microscale, muscle comprises a collection of parallel nerve-activated fibers, which transition into passive collagen-based fibers of the tendon through the endomysium. This transition is characterized by actin microfilaments forming finger-like projections into the tendinous extracellular matrix [8]. Analogous to skeletal MTJs, transitions from muscle to tendons in cardiac tissues are localized in the connections between the papillary muscles (PM), and the chordeae tendineae (CT) in the left and right ventricles. The CT are connected to the LL to prevent LL folding and blowing out over themselves during a heart cycle. Structurally, the CT–LL transition is characterized by a transition of two different arrangements of fibrillar collagen and proteoglycan (PG), while the CT–PM transition consists chiefly of fibrillar collagen and PG blending into the PM [9] which exhibits dissimilar sarcomere lengths compared to other cardiac tissue [10].

### 1.2. Prior Work in Constitutive Modeling of Anisotropic Soft-Tissue

Constitutive modeling techniques in the literature for anisotropic soft-tissue are briefly reviewed next, with an emphasis on cardiac tissue. Broadly, continuum-based constitutive models can be classified into (a) phenomenological models and (b) structural models. Phenomenological models typically utilize anatomically relevant empirical observations without an explicit consideration for the microstructural contribution of the tissue constituents. For instance, transversely isotropic (TI) phenomenological models aggregate the hierarchical fiber microstructure into a lumped fiber representation inside an isotropic matrix [11]. These approaches have been used to model mitral valve (MV) mechanics [12,13], biaxial behavior of the myocardium [14] and bio-prosthetic human valves [15]. Additionally, orthotropic phenomenological models have been proposed using an anisotropic variant of the Fung-type hyperelasticity [16,17], and these models have been applied to the myocardial architecture, which has been shown to consist of sheets of fibers separated by cleavage planes [18].

In contrast to phenomenological models, structural models take into account the microstructural, compositional and hierarchical characteristics of the tissue. Inclusion of statistically distributed fibers in anisotropic tissue has been shown in the structural modeling of aortic valves [19,20,21] and mitral valves [22,23]. Structural models accounting for the sheet-based layered microstructure of the myocardium have been presented in [24,25]. Another structural model with a distributed collagen fiber network in hierarchical arterial layers is shown in [26]. A similar approach of piecewise modeling of constituent cardiac tissue is presented in [27,28]. For a comprehensive review of biomechanical continuum modeling of cardiac tissue, readers are referred to [11,29,30].

In light of the discussion in Section 1.1 indicating gradual changes in the tissue microstructure and composition in the transition regions, there appears a need to account for the tissue transition morphology in the constitutive model, which thus far has primarily focused on the hierarchical makeup of tissue constituents [25,26,27,28], as discussed above. Early work in functional grading (gradual spatial change in material properties) of cardiac tissue can be found in the works of Chen et al. [31], where a TI model was used to account for the CT–LL transition, and the fiber orientations and the isotropic matrix parameter in the TI strain energy density were assumed to vary along the CT–LL transition. Recently, Rego et al. have proposed a functionally graded structural constitutive model to account for transmural variations in the collagen composition and fiber orientation in the LL of porcine aortic valves [32].

With this collective prior research in mind, it is important to state their applicability within the context of simulators at the organ level. Typically, a surgical simulator (or whole-body FE model) involves macroscale anatomical features, implying that any modeling approach accounting for tissue transition has to be scaled up to the macroscale. This presents an additional challenge of model validation and scalability-constitutive models of the transition properties developed based on microscale data need to be placed in context of load distribution characteristics that are likely to exist at the macroscale. In the case of cardiac simulators, for instance, there is a need to equip existing macroscale heart simulators with integrated fluid–structure interaction (FSI) at the organ/organelle level [22,33,34], with accurate constitutive material modeling capabilities at the cardiac tissue transitions at the microscale [31,32] to enhance their anatomical realism.

It should also be emphasized here that most surgical simulators/whole-body injury prediction tools currently assign phenomenological models to each macroscale organ/organelle [3,35,36,37]. As such, incorporation of structural models to account for tissue transitions [32] in surgical simulators/ whole-body injury prediction tools presents an immediate integration and compatibility challenge—the parameters of the structural model need re-calibration for backwards compatibility, particularly for those tissue regions that are currently assigned homogeneous phenomenological material properties. While structural models can offer potentially greater insights into the underlying tissue microstructure and function, nevertheless, an approach to modify parameters of existing phenomenological models in the tissue transition regions without reformulating the constitutive model to account for the microstructural characteristics in the transition regions is particularly attractive, especially within the context of surgical simulators/whole-body injury prediction tools.

### 1.3. Problem Statement and Scope

**Figure 1:**
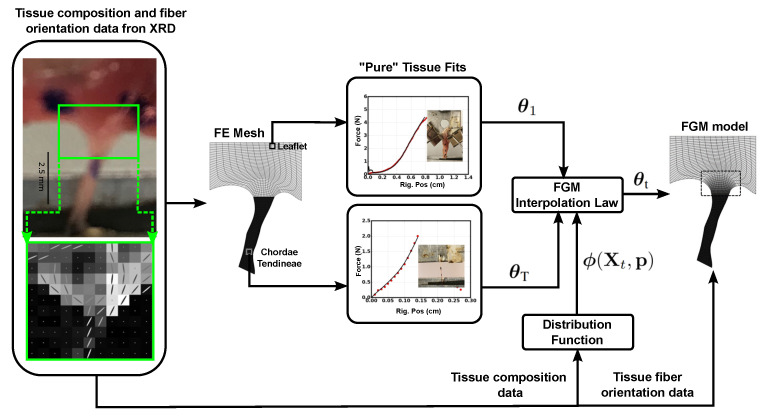
Overview of the proposed functionally graded material (FGM) modeling framework.

In this work, we report on the development of an approach for functional grading of a phenomenological TI hyperelastic model capable of replicating macroscale deformation, while being informed by high-resolution tissue composition and primary fiber orientation data (see Figure 1). We focus on the TI formulation originally proposed by Weiss et al. [38], which admits an exponential toe region followed by a linear extension regime. Our choice of this TI formulation (henceforth in this work, the term “TI” refers to the Weiss et al. [38] formulation of transverse isotropy) is governed by (a) its widespread utility in modeling anisotropic soft tissue such as such as knee ligaments [38,39], fascia lata [40] and Achilles tendon [41], and (b) its availability in existing commercial finite element solvers such as LS-DYNA [42] and FEBio [43]. There are two key sub-problems addressed within the scope of this work:

1. *FGM interpolation problem for the TI model:* Given the TI model fits represented by $\theta_{1}$ and $\theta_{T}$ for the terminal materials at either side of the transition which are assumed to be homogeneous (“pure” tissue), and a user-defined distribution function $\phi (X_{t},p)$ represented by shape parameters p, formulate an interpolation law to obtain functionally graded TI parameters $\theta_{t}$ at transition layer *t* with coordinates given by $X_{t}$, where t=1,⋯,T.
2. *Shape parameter estimation problem:* Given experimental data on the constituent composition and molecular strain at the transition region, obtain the optimal shape parameter p of the distribution function $\phi (X_{t},p)$ using optimization techniques.

We assume that the deformation characteristics of both “pure” tissue and their transition regions display transversely isotropic behavior adequately described by the TI formulation by Weiss et al. [38]. Previous experimental studies in our group showed that using a combination of optical microscopy and XRD imaging techniques [44], dissimilar mechanical responses could be observed in CT–LL and CT–PM transition regions in porcine tricuspid valve (TV) and mitral valve (MV) compared to the the “pure” CT, LL and PM tissue under load. In this work, we utilize these experimental results to characterize the CT–LL and CT–PM transition regions by solving the aforementioned two sub-problems (see Figure 1). In this work, predictions of the collagen fiber orientations and the strain distribution in the transition regions under tensile load using a FE implementation of our approach are validated against XRD measurements. All simulation results showed good agreement with experimental data.

In the subsequent Materials and Methods section, the theoretical details of the functionally graded TI model, the proposed FGM interpolation law (sub-problem 1), the experimental protocol for model validation and an FE implementation with the associated parameter estimation (sub-problem 2) are discussed. Next, in the Results section, the proposed model is validated on uniaxial tension tests on CT–LL and CT–PM transition regions of porcine heart valves. Finally, we conclude with the discussion of our approach and future work.

## 2. Materials and Methods

### 2.1. FGM Modeling

#### 2.1.1. TI Model Constitutive Law

In this section, the constitutive stress–strain relationship of the TI model is briefly reviewed. The decoupled strain energy density for the TI model is given by [38]:

(1) $$\begin{array}{ccc} W & = & \tilde{W}\left(\tilde{C}\right)+U\left(J\right)\\ & = & \underset{\mathrm{isotropic}}{\underset{︸}{{\tilde{F}}_{1}\left({\tilde{I}}_{1},{\tilde{I}}_{2}\right)}}+\underset{\mathrm{fiber}}{\underset{︸}{{\tilde{F}}_{2}\left({\tilde{I}}_{4}\right)}}+\underset{\mathrm{volumetric}}{\underset{︸}{U(J)}} \end{array}$$

where *J* is the Jacobian of the deformation, ($\tilde{\cdot }$) indicates the deviatoric component of the terms in parenthesis, and $F_{1}$, $F_{2}$ and *U* indicate the strain energy density components pertaining to the isotropic, fiber and the volumetric contributions, respectively. The volumetric strain energy density U(J) and the invariants ${\tilde{I}}_{l}$ (where l∈{1,2,4}) of the right deviatoric Cauchy–Green strain tensor $\tilde{C}$ are given by:

(2a) $$\begin{array}{c} U=\frac{K}{2}{\left(\ln J\right)}^{2} \end{array}$$

(2b) $$\begin{array}{c} {\tilde{I}}_{1}=\mathrm{tr}\tilde{C}=J^{-2/3}\mathrm{tr}C \end{array}$$

(2c) $$\begin{array}{c} {\tilde{I}}_{2}=\frac{1}{2}\left({\left(\mathrm{tr}\tilde{C}\right)}^{2}-\mathrm{tr}{\tilde{C}}^{2}\right) \end{array}$$

(2d) $$\begin{array}{c} {\tilde{I}}_{4}=a_{0}^{T}\tilde{C}a_{0}=J^{-2/3}a_{0}^{T}Ca_{0} \end{array}$$

where *K* is the bulk modulus and $a_{0}$ is the unit vector along the fiber orientation in the undeformed configuration.

The Cauchy stress can be derived from Equation (1) as follows [38]:

(3) $$\begin{array}{c} \sigma =p1+\frac{2}{J}\left[\left({\tilde{W}}_{1}+{\tilde{W}}_{2}{\tilde{I}}_{1}\right)\tilde{B}-{\tilde{W}}_{2}{\tilde{B}}^{2}+{\tilde{W}}_{4}{\tilde{I}}_{4}a⊗a-\frac{1}{3}\left({\tilde{W}}_{1}{\tilde{I}}_{1}+2{\tilde{W}}_{2}{\tilde{I}}_{2}+{\tilde{W}}_{4}{\tilde{I}}_{4}\right)1\right] \end{array}$$

where ${\tilde{W}}_{l}=\partial \tilde{W}/\partial {\tilde{I}}_{l}$, *p* is the hydrostatic pressure given by p=∂U/∂J, a is the unit vector in the fiber orientation in the deformed configuration, B is the left Cauchy–Green strain tensor and 1 is the identity tensor. The unit vector a in the deformed configuration can be related to the undeformed fiber orientation $a_{0}$ by the deformation gradient tensor F as follows:

(4) $$\begin{array}{c} \lambda a=Fa_{0} \end{array}$$

where λ is the magnitude of fiber stretch.

The strain energy function for the TI model uses a Mooney–Rivlin hyperelastic formulation for the the isotropic terms and a multi-regime strain energy formulation for the fiber terms, as given below [38]:

(5) $$\begin{array}{c} {\tilde{F}}_{1}\left({\tilde{I}}_{1},{\tilde{I}}_{2}\right)=C_{1}\left({\tilde{I}}_{1}-3\right)+C_{2}\left({\tilde{I}}_{2}-3\right) \end{array}$$

$$\begin{array}{c} \tilde{\lambda }\frac{\partial {\tilde{F}}_{2}}{\partial \tilde{\lambda }}=\left\{\begin{array}{cc} 0 & \tilde{\lambda }\leq 1\\ C_{3}\left(e^{C_{4}\left(\tilde{\lambda }-1\right)}-1\right) & 1\leq \tilde{\lambda }<\lambda^{*}\\ C_{5}\tilde{\lambda }+C_{6} & \tilde{\lambda }\geq \lambda^{*} \end{array}\right. \end{array}$$

where $\tilde{\lambda }$ is the deviatoric fiber stretch given by:

(6) $$\begin{array}{c} \tilde{\lambda }=\sqrt{{\tilde{I}}_{4}} \end{array}$$

**Figure 2:**
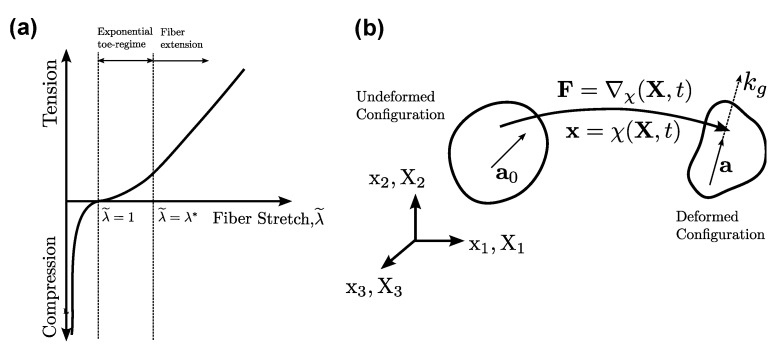
(**a**) Regime-dependent nature of the transversely isotropic (TI) model and (**b**) schematic of the continuum representation of the TI model directionality.

As seen from Equation (5), seven hyperelastic parameters $\theta ={\left[C_{1},C_{2},C_{3},C_{4},\lambda^{*},C_{5},C_{6}\right]}^{T}$ completely characterize the deviatoric stress response of the TI model. The influence of each of these parameters is enumerated as follows:

1. $C_{1}$, $C_{2}$: These correspond to the isotropic stiffness of the matrix component of the material.
2. $C_{3}$, $C_{4}$, $\lambda^{*}$: These correspond to the fiber component of the material and are used to model the exponential toe region of the stress–strain curve. $C_{3}$ and $C_{4}$ control the degree of exponential rise, while the critical fiber stretch, $\lambda^{*}$, controls the extent of the exponential regime (see Figure 2a).
3. $C_{5}$, $C_{6}$: These correspond to the post-exponential linear stretching of the fiber. $C_{5}$ represents the modulus of straightened fibers.

From Equation (5), it can be seen that the stress enhancement due to the embedded fibers is only active in tension $\tilde{\lambda }>1$.

It should be noted herein that in Weiss’s formulation [38], $C_{6}$ is dependent on $C_{3}$–$C_{5}$ and $\lambda^{*}$ due to enforcement of $C^{0}$ continuity at $\tilde{\lambda }=\lambda^{*}$. In addition, if $C^{1}$ continuity is assumed, $C_{5}$ drops out as an independent variable as well, leading to the following equations:

(7a) $$\begin{array}{cc} C_{5}= & C_{3}C_{4}e^{C_{4}\left(\lambda^{*}-1\right)} \end{array}$$

(7b) $$\begin{array}{cc} C_{6}= & C_{3}\left(e^{C_{4}\left(\lambda^{*}-1\right)}-1\right)-C_{5}\lambda^{*} \end{array}$$

The tensorial representation of the elasticity in the undeformed configuration is given by $4\partial^{2}W/\partial C\partial C$. For the purposes of functional grading, some notion of the the instantaneous stiffness along the fiber orientation is desired. We define a scalar metric called the *grading stiffness*, $k_{g}$, to be the rate of change of the traction stress vector along the fiber orientation a, as shown below (see Figure 2b):

(8) $$\begin{array}{cc} k_{g} & ≜\frac{\partial }{\partial \tilde{\lambda }}\left(a^{T}\sigma a\right) \end{array}$$

Using the expression for the Cauchy stress from Equation (3), the grading stiffness, $k_{g}$, can be written as:

(9) $$\begin{array}{cc} k_{g}=\frac{\partial }{\partial \tilde{\lambda }}[\frac{2}{J}[\left({\tilde{W}}_{1}+{\tilde{W}}_{2}{\tilde{I}}_{1}\right)a^{T}\tilde{B}a- & {\tilde{W}}_{2}a^{T}{\tilde{B}}^{2}a-\frac{1}{3}\left({\tilde{W}}_{1}{\tilde{I}}_{1}+2{\tilde{W}}_{2}{\tilde{I}}_{2}\right)\\ & +{\tilde{W}}_{4}{\tilde{I}}_{4}\left(a^{T}\left(a⊗a\right)a-\frac{1}{3}\right)]+\frac{K\ln J}{J}] \end{array}$$

Soft tissues are generally assumed nearly-incompressibility due to high levels of hydration, due to which it can be assumed that J≈1 and $\partial J/\partial \tilde{\lambda }\approx 0$. From the Caley–Hamilton theorem, it can be shown that: ${\tilde{B}}^{2}={\tilde{I}}_{1}\tilde{B}-{\tilde{I}}_{2}1+J^{-1}{\tilde{B}}^{-1}$. The final expression of $k_{g}$ is thus obtained by assembling the hyperelastic parameters from Equation (9), as given below:

(10) $$\begin{array}{c} k_{g}\approx C_{1}\gamma_{1}\left(\tilde{\lambda }\right)+C_{2}\gamma_{2}\left(\tilde{\lambda }\right)+\frac{2}{3}\frac{\partial }{\partial \tilde{\lambda }}\left[\tilde{\lambda }\frac{\partial {\tilde{F}}_{2}}{\partial \tilde{\lambda }}\right] \end{array}$$

where $\tilde{\lambda }\partial F_{2}/\partial \tilde{\lambda }$ is given by Equation (5) and $\gamma_{1}\left(\tilde{\lambda }\right)$ and $\gamma_{2}\left(\tilde{\lambda }\right)$ are deformation dependent terms given by:

(11) $$\begin{array}{cc} \gamma_{1}\left(\tilde{\lambda }\right)= & 2\frac{\partial a^{T}\tilde{B}a}{\partial \tilde{\lambda }}-\frac{2}{3}\frac{\partial {\tilde{I}}_{1}}{\partial \tilde{\lambda }}\\ \gamma_{2}\left(\tilde{\lambda }\right)= & \frac{2}{3}\frac{\partial {\tilde{I}}_{2}}{\partial \tilde{\lambda }}-2\frac{\partial a^{T}{\tilde{B}}^{-1}a}{\partial \tilde{\lambda }} \end{array}$$

In the special case where the isotropic matrix is considerably less stiff than the fiber ($C_{1},C_{2}<<C_{5}$), $k_{g}\approx \left(2/3\right)C_{5}$ in the regime $\tilde{\lambda }>\lambda^{*}$ (see Equations (5) and (10)).

#### 2.1.2. Functional Grading of the TI Model

The proposed functional grading of the TI model seeks to obtain an expression for $\theta_{t}$ given by:

(12) $$\begin{array}{c} \theta_{t}={\left[{\left[C_{1}\right]}_{t},{\left[C_{2}\right]}_{t},{\left[C_{3}\right]}_{t},{\left[C_{4}\right]}_{t},{\left[C_{5}\right]}_{t},{\left[C_{6}\right]}_{t},{\left[\lambda \right]}_{t}^{*}\right]}^{T} \end{array}$$

across T discrete material layers, where t=1,⋯,T, subject to design constraints in the corresponding grading stiffness, ${\left[k_{g}\right]}_{t}$. The terminal materials fits are given by $\theta_{1}$ and $\theta_{T}$. It is assumed that $\theta_{1}$ and $\theta_{T}$ are available beforehand.

Using a distribution function $\phi (p,X_{t})$, and $\phi :R^{3}\to \left[0,1\right]$ to indicate the constituent fraction, the grading stiffness at each transition layer *t* can be written as:

(13) $$\begin{array}{c} {\left[k_{g}\right]}_{t}={\left[k_{g}\right]}_{1}+\left({\left[k_{g}\right]}_{T}-{\left[k_{g}\right]}_{1}\right)\phi \left(p,X_{t}\right) \end{array}$$

where $X_{t}$ refers to set of all material points belonging to transition layer *t* and p is a vector of shape parameters. As will be demonstrated later in Section 2.4, $\phi (p,X_{t})$ is informed by experimental data to create the FGM model. By definition, $\phi (p,X_{1})=0$, $\phi (p,X_{T})=1$. For considerations of simplicity, the distribution function $\phi (p,X_{t})$ is henceforth denoted by ϕ(t), unless stated otherwise.

For single parameter material models, the grading stiffness is a function of a single material parameter (such as the elastic modulus in linear elastic [45] or the shear modulus in neo-Hookean [46] material model). In the case of the TI model, however, this process is complicated due to (a) nonlinearity in its parameters and (b) multi-regime anisotropic nature of its formulation, as seen in the expression for $k_{g}$ in Equation (10). If the hyperelastic parameters $C_{4}$ and $\lambda^{*}$ are equal in both terminal materials, the grading stiffness in each transition layer can be explicitly written as follows:

(14) $$\begin{array}{c} {\left[k_{g}\right]}_{t}\approx \left\{\begin{array}{cc} {\left[C_{1}\right]}_{t}\gamma_{1}\left(\lambda \right)+{\left[C_{2}\right]}_{t}\gamma_{2}\left(\lambda \right)\; & \tilde{\lambda }\leq 1\\ {\left[C_{1}\right]}_{t}\gamma_{1}\left(\lambda \right)+{\left[C_{2}\right]}_{t}\gamma_{2}\left(\lambda \right)+\frac{2}{3}{\left[C_{3}\right]}_{t}{\left[C_{4}\right]}_{1}e^{{\left[C_{4}\right]}_{1}\left(\tilde{\lambda }-1\right)} & 1\leq \tilde{\lambda }<{\left[\lambda^{*}\right]}_{1}\\ {\left[C_{1}\right]}_{t}\gamma_{1}\left(\lambda \right)+{\left[C_{2}\right]}_{t}\gamma_{2}\left(\lambda \right)+\frac{2}{3}{\left[C_{5}\right]}_{t} & \tilde{\lambda }\geq {\left[\lambda^{*}\right]}_{1} \end{array}\right. \end{array}$$

where ${\left(\cdot \right)}_{t}={\left(\cdot \right)}_{1}+\left({\left(\cdot \right)}_{T}-{\left(\cdot \right)}_{1}\right)\phi \left(t\right)$.

The results of Equation (15) indicate that nonlinearities in the grading stiffness are restricted to parameters $C_{4}$ and $\lambda^{*}$. This implies that the TI parameters $C_{1}-C_{3}$ and $C_{5}$ can be individually interpolated using ϕ(t) while maintaining the corresponding distribution in the grading stiffness $k_{g}$, provided that $C_{4}$ and $\lambda^{*}$ are constant in the grading process.

However, constraining the terminal material $C_{4}$ and $\lambda^{*}$ in the grading process is not always desirable because it can compromise the accuracy of the terminal fits (An example of this case is presented in the experimental evaluation of the FGM model at the CT–LL transition regions of the TV in Section 2.4.1, where the CT and LL fits have dissimilar $C_{4}$ and $\lambda^{*}$). At the same time, interpolating $C_{4}$ and $\lambda^{*}$ eliminates the linear structure of the expression for $k_{g}$. However, we note here most applications of the TI model (and other hyperelastic models, in general) involve large deformations extending into the linear regime of the fiber characterized by $\tilde{\lambda }>\lambda^{*}$. In the deformation regime characterized by $\tilde{\lambda }>\max\left({\left[\lambda^{*}\right]}_{1},{\left[\lambda^{*}\right]}_{T}\right)$, the fiber component of $k_{g}$ is dependent on $C_{5}$, which it varies linearly with. This implies that if the critical fiber stretch can be interpolated monotonically as follows:

(15) $$\begin{array}{c} {\left[\lambda^{*}\right]}_{t}={\left[\lambda^{*}\right]}_{1}+\left({\left[\lambda^{*}\right]}_{T}-{\left[\lambda^{*}\right]}_{1}\right)\psi \left(t\right) \end{array}$$

where ψ(t) is a monotonic function, the desired distribution ϕ(t) in $k_{g}$ in the transition regime can be maintained in the regime $\tilde{\lambda }>\max\left({\left[\lambda^{*}\right]}_{1},{\left[\lambda^{*}\right]}_{T}\right)$.

Next, the FGM process for the the parameter $C_{4}$ is described. In order to quantify the discontinuity at the exponential-linear interface, we define a *discontinuity index*, *f*:

(16) $$\begin{array}{c} f≜\frac{C_{5}}{C_{3}C_{4}e^{C_{4}\left(\lambda^{*}-1\right)}} \end{array}$$

When $C^{1}$ continuity is preserved at $\tilde{\lambda }=\lambda^{*}$, f=1. However, in the Weiss TI model, $C^{1}$ continuity is not assumed, due to which the discontinuity index *f* may be dissimilar in the terminal material fits, i.e., ${\left[f\right]}_{1}\neq {\left[f\right]}_{T}\neq 1$. To ensure that the the discontinuity at the exponential–linear interface remains bounded, the discontinuity index *f* is subsequently interpolated using a monotonic function ψ(t), as given below:

(17) $$\begin{array}{c} {\left[f\right]}_{t}={\left[f\right]}_{1}+\left({\left[f\right]}_{T}-{\left[f\right]}_{1}\right)\psi \left(t\right) \end{array}$$

This process ensures discontinuity at the exponential–linear interface remains bounded, i.e., ${\left[f\right]}_{t}\in \left[{\left[f\right]}_{1},{\left[f\right]}_{T}\right]$, and sharp material stiffness discontinuities do not occur at this interface. The hyperelastic parameter ${\left[C_{4}\right]}_{t}$ can then be computed by solving the following nonlinear equation:

(18) $$\begin{array}{c} g\left({\left[C_{4}\right]}_{t}\right)≔{\left[f\right]}_{t}{\left[C_{3}\right]}_{t}{\left[C_{4}\right]}_{t}e^{{\left[C_{4}\right]}_{t}\left({\left[\lambda^{*}\right]}_{t}-1\right)}-{\left[C_{5}\right]}_{t}=0 \end{array}$$

The remaining parameter ${\left[C_{6}\right]}_{t}$ is computed by enforcing $C^{0}$ continuity in Equation(7b) at $\tilde{\lambda }=\lambda^{*}$.

#### 2.1.3. Interpolation Law for Functional Grading of TI Model

The discussions in the preceding sections are used as a basis for proposing a general interpolation law for functional grading of the TI model (Algorithm 1).

The inputs to the interpolation law for T−2 transition layers consist of the terminal material fits $\theta_{1}$, $\theta_{T}$, a vector of shape parameters p used to define the distribution function ϕ(t), a monotonic function ψ(t) to interpolate the parameter $\lambda^{*}$ and the discontinuity index *f*, and the maximum stretch used in the terminal fits, given by ${\left[\lambda_{max}\right]}_{1}$, ${\left[\lambda_{max}\right]}_{T}$. The output from the interpolation law are the graded properties $\theta_{t}$ for t=1,⋯,T. Based on the discussions in Section 2.1.2, the steps of the algorithm are self-evident, except two special cases, which are elaborated next.

Firstly, it may so happen that one or both of the terminal materials fits are obtained from experimental data where the fibers were still uncrimping in the exponential regime ($\lambda_{\max}<\lambda^{*}$) and linear fiber extension regime of the TI model was not utilized in the fitting process. Since the underlying experimental data in these fits did not capture the fiber extension phase characterized by $C_{5}$, it is likely that the fitting process would be insensitive to variations fitted terminal $C_{5}$. Functional grading based on ill-defined $C_{5}$ could potentially lead to spurious grading results. To account for these fitting scenarios, the $C_{5}$ term in those terminal fits corresponding to uncrimped fibers is recomputed to enforce $C^{1}$ continuity using Equation (16) with the discontinuity index f=1. This process ensures that the interpolation law is independent of the underlying experimental data or optimization framework used to obtain the terminal fits. Lines 7–18 of Algorithm 1 address this particular scenario. For well defined $C_{5}$, the process outlined in the preceding section suffices (lines 20–22 of Algorithm 1).
**Algorithm 1** FGM Interpolation Law for the TI model 1:**procedure**$\theta_{t}$=GenerateFGM($\theta_{1}$, $\theta_{T}$,T,p, ψ(t), ${\left[\lambda_{\max}\right]}_{1}$, ${\left[\lambda_{\max}\right]}_{T}$ ) 2:    ### **Grade Matrix Properties** ### 3:    ${\left[C_{1}\right]}_{t}\leftarrow \mathrm{interpolate}\left({\left[C_{1}\right]}_{1},{\left[C_{1}\right]}_{T},T,p\right)$, ${\left[C_{2}\right]}_{t}\leftarrow \mathrm{interpolate}\left({\left[C_{2}\right]}_{1},{\left[C_{2}\right]}_{T},T,p\right)$ 4:    ### **Grade Fiber Properties** ### 5:    ${\left[C_{3}\right]}_{t}\leftarrow \mathrm{interpolate}\left({\left[C_{3}\right]}_{1},{\left[C_{3}\right]}_{T},T,p\right)$ 6:    ${\left[\lambda^{*}\right]}_{t}\leftarrow \mathrm{interpolate}\left({\left[\lambda^{*}\right]}_{1},{\left[\lambda^{*}\right]}_{T},T,\psi \left(t\right)\right)$ 7:    **if** ${\left[\lambda_{\max}\right]}_{1}$<${\left[\lambda^{*}\right]}_{1}$ **and** ${\left[\lambda_{\max}\right]}_{T}$≥${\left[\lambda^{*}\right]}_{T}$ **then** 8:        ### **Terminal Material 1 fibers still uncrimping (exponential regime)** 9:        ${\left[f\right]}_{1}$ ← 110:        Recompute ${\left[C_{5}\right]}_{1}$ according to Equation (16).11:    **else if** ${\left[\lambda_{\max}\right]}_{T}$<${\left[\lambda^{*}\right]}_{T}$ **and** ${\left[\lambda_{\max}\right]}_{1}$≥${\left[\lambda^{*}\right]}_{1}$ **then**12:        ### **Terminal Material T fibers still uncrimping (exponential regime)**13:        ${\left[f\right]}_{T}$ ← 114:        Recompute ${\left[C_{5}\right]}_{T}$ according to Equation (16).15:    **else if** ${\left[\lambda_{\max}\right]}_{T}$<${\left[\lambda^{*}\right]}_{T}$ **and** ${\left[\lambda_{\max}\right]}_{1}$<${\left[\lambda^{*}\right]}_{1}$ **then**16:        ### **Both Terminal Material fibers still uncrimping (exponential regime)**17:        ${\left[f\right]}_{1}$ ← 1, ${\left[f\right]}_{T}$ ← 118:        Recompute ${\left[C_{5}\right]}_{1}$ and ${\left[C_{5}\right]}_{T}$ according to Equation (16).19:    **else**20:        ### **Both Terminal Material 1 and T fibers extending (linear regime)**21:        ${\left[f\right]}_{1}$ ← ${\left[C_{5}\right]}_{1}/{\left[C_{3}\right]}_{1}{\left[C_{4}\right]}_{1}e^{{\left[C_{4}\right]}_{1}\left({\left[\lambda^{*}\right]}_{1}-1\right)}$22:        ${\left[f\right]}_{T}$ ← ${\left[C_{5}\right]}_{T}/{\left[C_{3}\right]}_{T}{\left[C_{4}\right]}_{T}e^{{\left[C_{4}\right]}_{T}\left({\left[\lambda^{*}\right]}_{T}-1\right)}$23:    ${\left[C_{5}\right]}_{t}\leftarrow \mathrm{interpolate}\left({\left[C_{5}\right]}_{1},{\left[C_{5}\right]}_{T},T,p\right)$24:    ${\left[f\right]}_{t}\leftarrow \mathrm{interpolate}\left({\left[f\right]}_{1},{\left[f\right]}_{T},T,\psi \left(t\right)\right)$25:    **Solve for** ${\left[C_{4}\right]}_{t}$: $g\left({\left[C_{4}\right]}_{t}\right){\left[f\right]}_{t}{\left[C_{3}\right]}_{t}{\left[C_{4}\right]}_{t}e^{{\left[C_{4}\right]}_{t}\left({\left[\lambda^{*}\right]}_{t}-1\right)}-{\left[C_{5}\right]}_{t}=0\;\forall t$26:    Compute ${\left[C_{6}\right]}_{t}$ using Equation (7b).27:    $\theta_{t}\leftarrow \left[{\left[C_{1}\right]}_{t},{\left[C_{2}\right]}_{t},{\left[C_{3}\right]}_{t},{\left[C_{4}\right]}_{t},{\left[C_{5}\right]}_{t},{\left[C_{6}\right]}_{t},{\left[\lambda^{*}\right]}_{t}\right]$28:    **return** $\theta_{t}$

Secondly, it may also happen that one of the terminal materials is isotropic, with an ill-defined discontinuity index *f* and critical fiber stretch $\lambda^{*}$. In these cases, these parameters are set to 1, and are subsequently graded using the distribution function ϕ(t).

**Figure 3:**
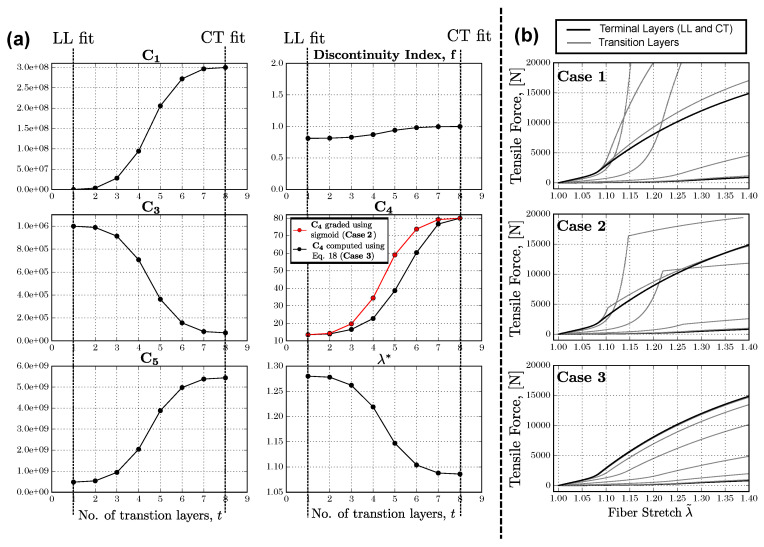
(**a**) Distribution of $C_{1},f,C_{3},C_{4},C_{5}$ and $\lambda^{*}$ in the chordea tendinea–leaflet (CT–LL) transition region of TV using the interpolation law proposed in Algorithm 1, used for the uniaxial tension tests in Case 3. Additionally overlaid in the distribution for $C_{4}$ is the corresponding graded parameter using a sigmoidal function, shown in red, corresponding to the $C_{4}$ distribution for Case 2. (**b**) Tensile force in the unit cell along the fiber orientation for Cases 1–3.

The efficacy of the proposed interpolation law is shown next using a unit cell tension study with six transition regions (T=8). The terminal material fits are chosen to be the LL and CT in TV specimens (see Table 1). For purposes of comparison, three case studies are described, as enumerated below:

1. **Case 1**. All TI parameters except $C_{5}$ are interpolated individually with ϕ(t) represented by a symmetric sigmoid (The explicit expression for the sigmoid is given later in Section 2.4.2—see Equation (21)). $C_{5}$ is computed by enforcing $C^{1}$ continuity at $\tilde{\lambda }=\lambda^{*}$ and using the other interpolated parameters.
2. **Case 2**. All TI parameters are interpolated using ϕ(t) used in Case 1.
3. **Case 3**: TI parameters interpolated with Algorithm 1 using ϕ(t), as in Cases 1 and 2. Without loss of generality, the function ψ(t) for interpolating the parameter $\lambda^{*}$ and the discontinuity index *f* is set to ϕ(t), since a sigmoid is by definition monotonic, i.e., ψ(t)≡ϕ(t). The resulting TI parameter distribution is shown in Figure 3a.

The tension response from the unit cells at each transition layer is shown in Figure 3b for Cases 1–3. It can be seen that the tensile responses for the transition layers all remain unbounded by those corresponding to the terminal fits (LL and CT) in Cases 1 and 2. More specifically, the tensile response in the transition regions in Case 1 shows enhanced stiffening behavior compared the upper-bounded response of the terminal material, whereas in Case 2, the tensile response is characterized by a sharp change at the $\tilde{\lambda }=\lambda^{*}$. Both these cases are highly undesirable, particularly because the underlying distribution function ϕ(t) was chosen to be smooth and monotonic. These effects are a direct consequence of the dissimilar $C_{4}$ and $\lambda^{*}$ in the LL and CT fits. In contrast, the tensile responses in Case 3 are bounded by the corresponding terminal responses with no sharp discontinuities in the tensile response.

In addition, shown in Figure 3b is the distribution of $C_{4}$ when it is independently interpolated (shown in red) using the sigmoidal function ϕ(t), which corresponding to the results of Case 2 in the unit cell study. It is noteworthy that while the distribution of $C_{4}$ in Cases 2 and 3 do not differ by much, the tensile response in the unit cell study is considerable, indicating a high sensitivity to the parameter $C_{4}$.

### 2.2. Experimental Methods

As discussed previously, the proposed FGM model is informed by (a) the terminal material TI fits acquired from tissue at either side of the transition which are assumed to be homogeneous (“pure” tissue) and (b) tissue composition data at the transition regions. Experimental characterization of the papillary muscle (PM), chordea tendinea (CT) and leaflet (LL) in excised porcine tricuspid valve (TV) and mitral valve (MV) and their transition regions were used to inform the FGM model as well as provide the experimental datasets for model validation. Due to the multiscale and anisotropic nature of these tissues with a primary fiber orientation [44], these anatomical regions are good candidates for development and validation of the proposed FGM model. The experimental protocol, setup and the actual datasets used in this work are presented in detail in our previous work [44]. An overview of the experimental procedures relevant to this study are briefly stated herein for purposes of completeness.

Uniaxial tensile tests were carried on the “pure” CT, LL and PM tissues, and the force–displacement data were fitted to the TI model to obtain the terminal fits. Details of the fitting procedure are described in Section 2.4.1.

**Figure 4:**
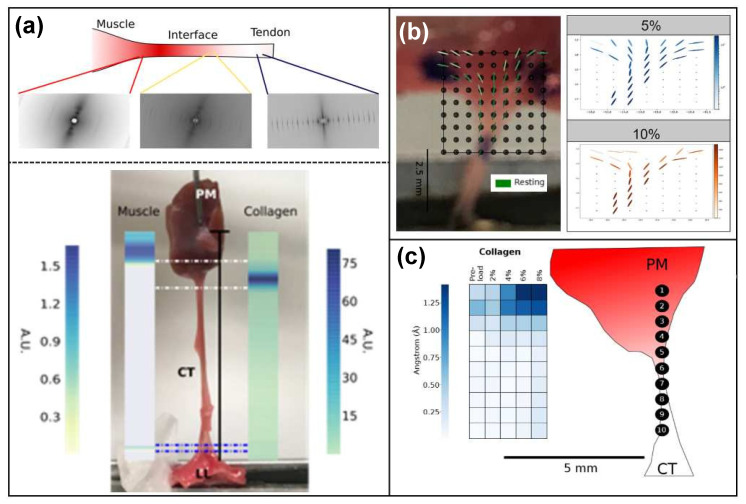
(**a**) XRD scanning process and relative tissue composition along CT–papillary muscle (PM) specimens, (**b**) collagen fiber orientation in the CT–LL specimens under 5% and 10% stretch and (**c**) D-period measurements in the CT–PM transition under load.

In addition to the conventional mechanical testing, X-Ray diffraction (XRD) imaging techniques were employed to obtain morphological and composition data about the tissue transition regions necessary to develop and validate the FGM models. Previous work in our group showed XRD imaging of musculoskeletal tissues resulted in unique diffraction patterns for “pure” muscle and “pure” tendon [47], while transition regions showed characteristics of both “pure” muscle and “pure” tendon diffraction patterns. Building upon these results, XRD imaging was utilized to investigate the relative tissue composition within the transition regions for the TV and MV specimens. Figure 4a shows the XRD scanning process and the relative tissue composition across the CT–PM transitions for a porcine MV specimen.

The collagen fiber orientation could also be obtained from processing of the XRD images [44], and these data were used in model development and validation in the CT–LL transition region where the local fiber orientation changes significantly between the CT and LL tissue (Figure 4b). Changes to the collagen fiber orientation under load were recorded for the CT–LL specimen to capture the localized tissue response for use in FGM model validation (described in Section 2.4.2).

Additionally, the XRD imaging process also provided a means to record local tissue response under load at the molecular level, which was used during the FGM model validation process for the CT–PM transition regions (described in Section 2.4.3). Tracking the local D-period change (Figure 4c) within the specimen under load provided a measure of the local strain distribution within the tissue, which was subsequently validated against the simulated strain distribution predicted by the FGM model.

### 2.3. Virtual Matched Pair FE Mesh Setup

The proposed FGM model was implemented numerically and an FE analysis approach was adopted to (a) characterize the “pure” CT, PM and LL tissue, and to (b) validate the FGM model at the CT–LL and CT–PM transitions using XRD data. The details of the FE mesh generation from the specimen geometry is discussed next.

Optical images of the experimental setup were imported into a computer aided design (CAD) program and scaled to the appropriate dimensions. Using the images as a guide, a solid model of the strain rig, load cell and tissue specimens were developed, which were then imported into a meshing tool and a solid hexahedral mesh was applied to the geometry using a commercial meshing software. This mesh development process was undertaken for the “pure” CT, PM and LL specimen tension simulations to determine the “pure” tissue fits, as well as the FGM model validation study in the CT–LL and CT–PM transition regions. Boundary conditions were explicitly modeled either using a rigid tie constraint or using contact formulations in the numerical solver between the tissue specimen and its attachment to the rig.

### 2.4. FE Simulation Setup and Parameter Estimation

Specimen-specific simulation details and the associated parameter estimation are described as follows:

#### 2.4.1. “Pure” CT, LL and PM Characterization

**Figure 5:**
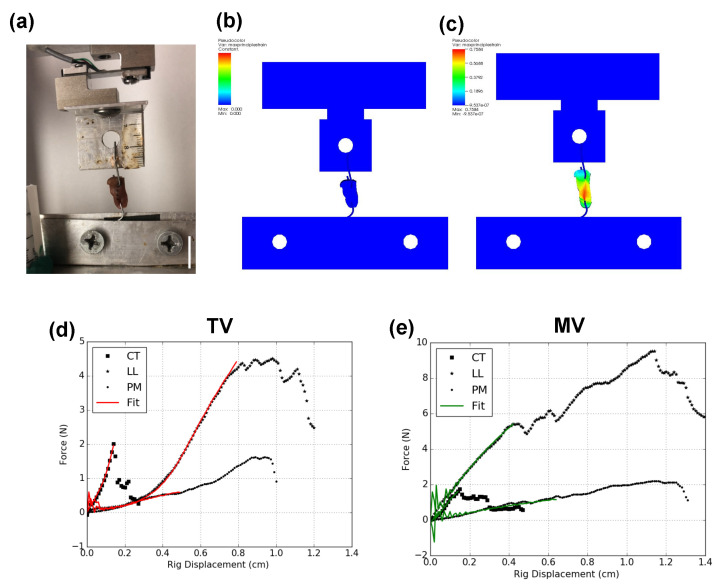
Uniaxial tension tests to characterize “pure” tissue specimens. (**a**) PM tissue in TV under uniaxial tension test, (**b**) corresponding virtual matched pair test, (**c**) strain distribution in specimen, (**d**) force–displacement data for CT, LL and PM in TV specimens with their overlaid fits and (**e**) force–displacement data for CT, LL and PM specimens in MV with overlaid fits. Scale bar in (**a**) represents 15 mm.

A displacement-controlled virtual matched pair simulation was set to replicate a uniaxial tension test along the predominant fiber orientation for the characterizing the individual specimens in porcine TV and MV. An inverse FE analysis was then carried out using a single objective genetic algorithm in DAKOTA [48] to fit the force–displacement experimental data to the TI model. The fitting process was limited to the deviatoric parameters of the TI model and a large bulk modulus of *K* = 1.464 × 10^8^ Pa was assumed, based on earlier fitting results from Pena et al. [39] where the TI model was used to characterize the human anterior cruciate ligament (ACL) knee ligament. This choice was governed by the range of the other TI deviatoric parameters of the ACL fit in [39], which were close to the fitted deviatoric TI parameters obtained in our study, leading to similar levels of slight compressibility in the numerical implementation. The fitted TI parameters for the CT, LL and PM specimens are given in Table 1. An example demonstrating the fitting process for the PM specimen of TV is shown in Figure 5 along with the optimal TI fits for all specimens.

#### 2.4.2. CT–LL transition

**Figure 6:**
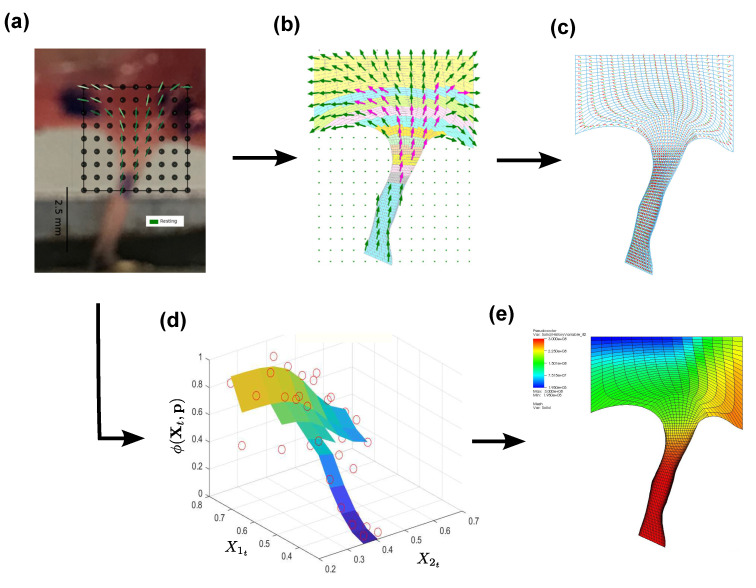
FGM model setup in CT–LL transition of TV. (**a**) XRD diffraction imaging producing fiber orientation and collagen intensity at grid points in the transition region, (**b**) fiber orientations mapped to a virtual matched pair finite element (FE) model, (**c**) fiber orientations assigned to the mesh on a per-element basis, (**d**) normalized intensity overlaid with fitted distribution function $\phi (X_{t},p)$ and (**e**) functionally graded parameter $C_{1}$ assigned on a per-element basis to the mesh using $\phi (X_{t},p)$ in (**d**). Scale bar in (**a**) represents 2.5 mm.

A virtual matched pair test was set up to develop and validate the FGM model to experimental data at the CT–LL transition under load. In addition to the specimen geometry, collagen fiber orientation and tissue composition details in the transition region were extracted from the XRD data (see Figure 6). The collagen fiber orientation was incorporated into the FE model using the following steps:

1. First, the experimental specimen frame was rigidly registered to the FE mesh of the CT–LL specimen.
2. After registration, the collagen orientations in the transition region obtained from XRD were mapped to the corresponding mesh by assigning to each element of the mesh the corresponding collagen orientation using a KD-tree based nearest-neighbor search.
3. Fiber orientations were subsequently propagated to the whole CT–LL mesh (regions outside of the scanned region of the specimen), by extrapolating the XRD orientation data.

Next, the relative diffraction signal intensity between the LL and CT constituents was used to parameterize the constituent distribution function ϕ(t) to generate the FGM model. Several smooth, monotonic distribution functions have been suggested to grade FGM in the literature; in particular, it has been reported that for unidirectional FGM applications, sigmoidally varying FGM leads to the reduced stress concentrations at the transition regions compared to linear, power and exponential distributions in transversely loaded linear elastic plates [45,49]. However, in the CT–LL regions of TV, it was found that the diffraction signal intensities varied both along, and transverse, to the CT–LL transition region, most likely due to the influence of a neighboring CT in the LL insertion. This led us to propose a bidirectional distribution function expressed as a product of two unidirectional monotonic functions with saturation, as shown below:

(19) $$\phi \left(X_{t},p\right)=\max(0,\min\left(1,\phi \left(X_{1_{t}},p\right)\phi \left(X_{2_{t}},p\right)\right)$$

where $\phi (X_{1_{t}},p)$ and $\phi (X_{2_{t}},p)$ are given by:

(20) $$\phi \left(X_{1_{t}},p\right)=p_{1}\left(\frac{e^{p_{2}\left(X_{1_{t}}-p_{3}\right)}-e^{-p_{2}\left(X_{1_{t}}-p_{3}\right)}}{e^{p_{2}\left(X_{1_{t}}-p_{3}\right)}-e^{-p_{2}\left(X_{1_{t}}-p_{3}\right)}}\right)+p_{4}$$

(21) $$\begin{array}{c} \phi \left(X_{2_{t}},p\right)=\left\{\begin{array}{cc} 0.50{\left(1+2{\bar{X}}_{2_{t}}\right)}^{p_{5}} & \mathrm{for}-0.5\leq {\bar{X}}_{2_{t}}\leq 0\\ 1-0.5{\left(1-2{\bar{X}}_{2_{t}}\right)}^{p_{6}} & \mathrm{for}\;0\leq {\bar{X}}_{2_{t}}\leq 0.5 \end{array}\right. \end{array}$$

where

(22) $${\bar{X}}_{2_{t}}=\frac{X_{2_{t}}-X_{2_{1}}}{X_{2_{T}}-X_{2_{1}}}-\frac{1}{2}$$

and $p={\left[p_{1},\cdots ,p_{6}\right]}^{T}$ is a vector of shape parameters. The distribution $\phi (X_{1_{t}},p)$ is a modified tan-hyperbolic function, used to model the relative intensity changes within the leaflet, while $\phi (X_{2_{t}},p)$ is a symmetric sigmoid $(\phi (X_{2_{t}},p)=0.5$ at ${\bar{X}}_{2_{t}}=0$, i.e., midway in the transition region), used for unidirectional FGM problems [45,49]. The shape parameters p were estimated using a simplex fitting algorithm to parameterize the distribution function, and the resulting surface fit overlaid with the relative intensity data from XRD is shown in Figure 6d. The interpolation law (Algorithm 1) outlined in Section 2.1.3 was then invoked to complete the FGM model. Since both $\phi (X_{1_{t}},p)$ and $\phi (X_{2_{t}},p)$ are monotonic, the parameter $\lambda^{*}$ and the discontinuity index *f* were graded using $\phi (X_{t},p)$. i.e., $\psi \left(t\right)\equiv \phi \left(X_{t},p\right)$. The interpolated TI parameters were then mapped onto the individual elements (Figure 6e) using a custom-developed keyword, ^*^STATE MATERIAL DEFINITION, implemented within Velodyne—the finite element solver used in this study (see Section 2.5).

#### 2.4.3. CT–PM Transition

The simulation setup for the FGM model implementation and validation on the CT–PM transition regions under load are described next. Due to the relatively large compliance of the PM specimens compared to the CT (see Table 1), and the observation that microtears are initiated at the CT–PM transition during uniaxial tension in whole LL–CT–PM specimen [44], the local strain distribution at the CT–PM transition was considered to be a strong determinant of the mechanical characteristics at this transition. Hence, the strain distribution in the transition region was used for validation against the simulation predictions from the FGM model.

**Figure 7:**
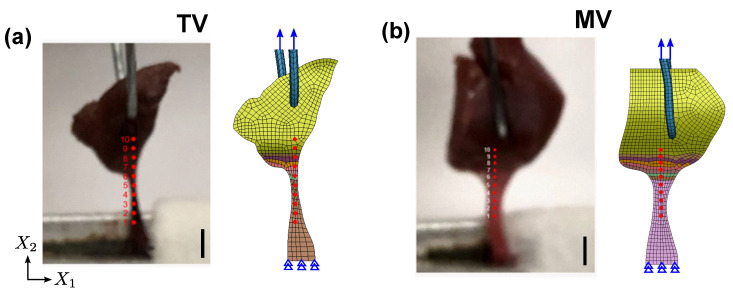
Virtual matched pair test setup for porcine (**a**) TV and (**b**) MV specimens with fiducial markers shown in red. Scale bar represents 3 mm.

The experimental strain information at the CT–PM transition was obtained as follows. Ten fiducial points along the CT–PM were selected along the sample centerline (see Figure 7a,b), while the specimen was under tensile load. The D-period change in fibrillar collagen at these ten locations were obtained by processing XRD images for each of these ten points under load (0–10% stretch), and the relative measure compared to the resting (no-load) configuration was used to denote the collagen molecular strain [47,50]. Similar to the simulation setup in Section 2.4.2, virtual matched pair tests were set up using the optical image of the specimen clamped in the strain rig, and the nodal coordinates in the FE model corresponding to the fiducials were tracked during the tension simulation to calculate the macroscale strain in the specimen. In contrast to the CT–LL specimens, the fiber orientation distribution and the collagen intensity distribution were not available for the CT–PM specimens, largely due to the small size of the transition region (∼2.7 mm) compared to the CT–LL transition (∼5.1 mm), which prevented an accurate extrapolated map of the fiber orientations and collagen intensity throughout the CT–PM transition. Consequently, the fiber orientations were aligned uniformly with the global $X_{2}$ direction. The constituent distribution function $\phi (X_{t},p)$ was assumed unknown and estimated using the molecular strain information. This methodology is described next.

Previous works have demonstrated that affine fibril kinematics (i.e., microscopic fiber motion follows macroscopic deformation) may be applied to model leaflet and the constituent collagen fibers [51,52,53]. Other experimental and molecular dynamics simulations have also described the nanomechanics of the various hierarchical levels of the fibrillar packing of collagen [54]. Specifically, it has been shown previously that a linear relationship exists between the D-period change in fibrillar collagen and the macroscopic engineering strain of the specimen at the linear extension regime of the collagen fibrils in rat tail tendons [55], and more recently in the porcine heart tissue transitions [44]. We utilize this observation to relate the molecular strain (expressed as percentage change in the collagen D-period) at the CT–PM transition to the engineering strain from the FE simulations to estimate the distribution function. Specifically, an inverse FE analysis was used to iterate on the shape parameters p at the CT–PM transition to minimize the squared residual between the normalized molecular and the normalized simulation strains at the fiducials, as shown below:

(23) $$\hat{p}=\underset{p}{\mathrm{argmin}}\sum_{i=0}^{i=n}{\left({\bar{\epsilon }}_{{exp}_{i}}-{\bar{\epsilon }}_{{sim}_{i}}\right)}^{2}$$

where ${\bar{\epsilon }}_{exp}$ and ${\bar{\epsilon }}_{sim}$ are normalized molecular strains and normalized engineering strains, respectively, obtained at *n* fiducials. It is worth mentioning here that while Fratzl et al. [55] reported a conversion factor of 0.4 between the molecular and macroscopic engineering strains, respectively, we chose normalized measures in Equation (23) to estimate the shape parameters, primarily to eliminate the effect of sample-to-sample variations in the mechanical properties of “pure” CT and PM used in the FGM model and those corresponding to the CT–PM specimen used in this study. In addition, the optimization step in Equation (23) was restricted to strains at the maximum specimen stretch (10%), in order to ensure that most of the fibrillar collagen in the CT–PM transition was in the extension regime (where the linear relationship between the molecular and macroscopic strain holds [55]).

In the CT–LL transition, a two-dimensional distribution function was used to interpolate the TI parameters due to experimental data that indicated existence of a transverse gradient in diffraction signal intensities in the LL; in contrast, no such data were available in the CT–PM. Furthermore, since the molecular strain was observed along the central axis of the CT, it is unlikely that any transverse asymmetry would be captured using the fiducials shown in Figure 7a,b. This led us to assume unidirectional grading along the $X_{2}$ direction in the CT–PM transition model, and a modified version of the distribution function from Equation (21) was proposed, as stated below:

(24) $$\begin{array}{c} \phi \left(X_{t},p\right)=\left\{\begin{array}{cc} \phi_{1}\left(X_{2_{t}},p\right)=\phi_{11}+\phi_{12}{\left(1+2{\bar{X}}_{2_{t}}\right)}^{w} & \mathrm{for}-0.5\leq {\bar{X}}_{2_{t}}\leq p_{1}\\ \phi_{2}\left(X_{2_{t}},p\right)=\phi_{21}+\phi_{22}{\left(1-2{\bar{X}}_{2_{t}}\right)}^{p_{2}} & \mathrm{for}\;p_{1}\leq {\bar{X}}_{2_{t}}\leq 0.5 \end{array}\right. \end{array}$$

subject to the following smoothness constraints:

(25) $$\begin{array}{cc} \; & \phi (-0.5,p)=0,\phi (0.5,p)=1\\ \; & \phi_{1}\left(p_{1},p\right)=\phi_{2}\left(p_{1},p\right)=0.5\\ \; & \phi_{1}^{\prime }\left(p_{1},p\right)=\phi_{2}^{\prime }\left(p_{1},p\right) \end{array}$$

where $p={\left[p_{1},p_{2}\right]}^{T}$ is a vector of shape parameters and ${\bar{X}}_{2_{t}}$ is given by Equation (22). The expression in Equation (24) can be considered a generalized version the Equation (21), which admits asymmetry in the sigmoidal shape. For known values of $p_{1}$ and $p_{2}$, the smoothness constraints in Equation (25) can be solved numerically to obtain the parameters *w* and $\phi_{\mathrm{ij}},i,j\in 1,2$.

The optimal shape parameters $\hat{p}$ are obtained by solving Equation (23) using a single-objective genetic algorithm using DAKOTA [48]. The transition properties were assigned to the mesh using Algorithm 1, and similar to the CT–LL transition case, we assumed $\psi \left(t\right)\equiv \phi \left(X_{t},p\right)$ since $\phi (X_{t},p)$ in Equation (24) is monotonic.

### 2.5. Numerical Implementation

The numerical implementation of aforementioned FE model was carried out in Velodyne v3.108, which is a massively parallel nonlinear FE solver developed by Corvid Technologies. Several core numerical schemes, such as single integration point solid elements, hourglass controls and central difference time integration parallels their counterparts in LS-DYNA and DYNA3D [42]. The contact force solver is based upon the slave–master formulation for node-segment contact, and is solved using a Lagrange multiplier approach to ensure that Karush–Kuhn–Tucker (KKT) inequality holds. Velodyne has been extensively utilized for investigation and validation of impact biomechanics problems [3,56] and underbody blast modeling [3]. For all simulations used in this study, an explicit FE solution was used.

## 3. Results

### 3.1. FGM Model Validation in CT–LL Transition

**Figure 8:**
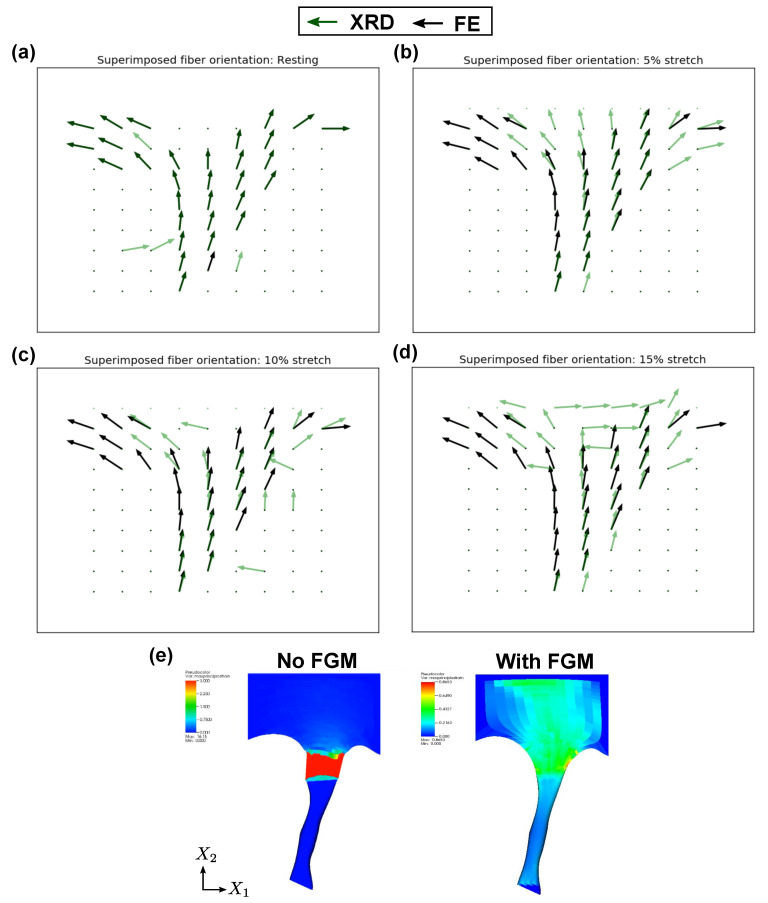
Local fiber orientation in the simulated CT–LL transition and the experimental data obtained from the analysis of the X-ray diffraction images in TV for (**a**) resting configuration, (**b**) 5% stretch, (**c**) 10% stretch and (**d**) 15% stretch. (**e**) Simulated strain distribution in the CT–LL transition in TV with hard transition without FGM (left) and with FGM (right) using Algorithm 1.

Simulation results of the CT–LL specimen in porcine TV under load are shown in Figure 8. The fiber orientations in the CT–LL specimen obtained from XRD measurements under load were compared with their corresponding simulation predictions. The superimposed fiber orientations obtained at resting (undeformed configuration), 5%, 10% and 15% stretch are shown in Figure 8a–d.

The fiber orientations at resting show a predominant longitudinal directionality near the “pure” CT end of the transition (Figure 8a), which progressively changes to the transverse direction near the LL insertion—an observation noted previously in [31]. Under load, the simulated fiber orientations show good agreements with XRD datasets Figure 8b–d. The simulated fiber stretch (magnitude of the orientation vectors) is also larger in regions closer to the LL than the CT, which is a consequence of greater compliance in the “pure” LL tissue compared to “pure” CT tissue, and this is reflected in the FGM model. In addition, certain outliers can be observed in the XRD orientations at 15% stretch which are near-orthogonal to the simulated orientations—these are most likely attributed to XRD signals from secondary fibers in the transition region [44].

For purposes of qualitative comparison, a tension simulation without the functional grading at the transition (hard transition in the CT and LL properties) is also shown in Figure 8e. While the hard transition case shows a pronounced necking leading to numerical instabilities due to the non-smooth material distribution, a smooth strain distribution can be observed using the FGM approach.

A similar validation strategy was adopted for CT–LL transition regions in MV, and good agreement was found between the experimental and the simulated fiber orientations under load. These results have not been included in this work for purposes of brevity.

### 3.2. FGM Model Validation in CT–PM Transition

**Figure 9:**
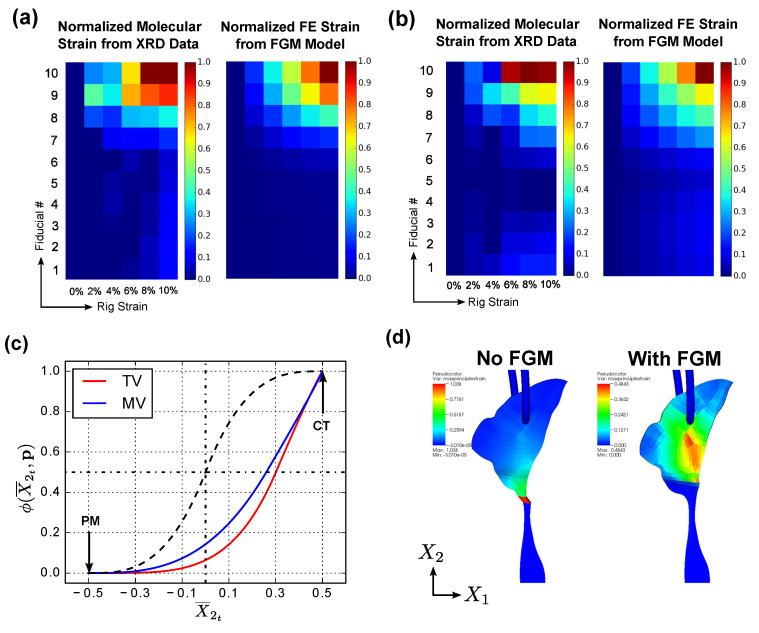
Colormaps indicating normalized strain distribution from XRD and inverse FE in the CT–PM transition regions for (**a**) TV and (**b**) MV specimens. (**c**) Optimized constituent distribution function $\phi (X_{2_{t}},p)$ with $\hat{p}=\left(0.301,1.084\right)$ and $\hat{p}=\left(0.26,0.945\right)$ for the TV and MV specimens, respectively. Additionally, overlaid in (**c**) is a symmetric sigmoid for purposes of comparison. (**d**) Strain distribution in CT–PM transition in TV with hard transition (no FGM) and with FGM using Algorithm 1.

Colormaps representing the normalized strain distribution (0–10% in increments of 2%) on the X-axis and the fiducial location on the Y-axis) are shown in Figure 9a,b for both the molecular strain and the macroscopic simulated strain. The corresponding optimal shape parameters $\hat{p}$ for TV and MV porcine specimens using the inverse FE solution discussed in Section 2.4.3 are shown in Figure 9c.

From Figure 9a,b, quantitative agreement can be found between normalized molecular strains and the macroscopic simulated strains. Despite the significantly reduced cross-section at the CT, most of the deformation is located closer to the more compliant PM in the transition region-indicating a complex interplay between geometric and material stiffness at the CT–PM transition regions. These results are also consistent with recent findings of rupture occurring on the PM-side of the CT–PM transition during uniaxial tension tests [44]. It is also noteworthy that the simulated strains agree most with the molecular strains for the 10% loading case, since these data were used in the inverse FE analysis for optimization of the shape parameters.

The rationale for the choice of an asymmetric sigmoidal shape in the constituent distribution function for the CT–PM transition regions becomes immediately apparent from the distribution profile in Figure 9c, which is characterized by a gradual change near the “pure” PM tissue followed by a steep rise near the CT insertion. The deviation of the profile from a symmetrical sigmoid is also apparent, which is shown overlaid in Figure 9c. These results indicate an asymmetric distribution of TI properties at the CT–PM transition for both TV and MV specimens, which is consistent with the experimental observation of a steep increase in collagen content near the CT in our previous work [44].

Qualitatively, the FGM model shows a smooth strain distribution compared to the hard transition case (no FGM) where numerical instabilities and necking at the CT are seen (Figure 9d), paralleling the observations in the CT–LL transition shown in Section 3.1.

## 4. Discussion

Despite increasing evidence suggesting unique morphological and biomechanical characteristics at tissue transitions and its implications in potential injury localization, there is limited research in the development of biomechanical models to replicate the deformation mechanics in surgical simulators and injury-prediction tools. This work attempts to address this research gap by presenting an FGM modeling approach wherein the transition properties are obtained as a function of the terminal material properties and material composition data at the transition region.

There are two main contributions of our FGM modeling work. First, an approach to interpolate the parameters of a phenomenological transversely isotropic (TI) model [38] was presented, which is characterized by nonlinearity and a multi-regime nature in its formulation. An algorithm for interpolating these parameters in order to ensure that the instantaneous stiffness in the fiber orientation obeys a user-defined spatial distribution was discussed. Canonical numerical implementations of the interpolation law using unit-cell tension studies (see Section 2.1.3) indicated that the tensile force in the transition layers could be bounded by the tensile force in the terminal materials in the large deformation regime.

Second, an approach to estimate the shape parameters of the distribution function using the XRD collagen intensity at the tissue-transitions was presented. An FE implementation of the proposed FGM model was carried out at the CT–LL and CT–PM tissue transitions in porcine TV and MV specimens, and the deformation characteristics were validated against experimental data. Specifically, the distribution function was informed directly from the collagen intensity in the CT–LL transition and the simulated fiber orientations were compared against the orientations obtained from XRD measurements under load (see Section 2.4.2). In the CT–PM transition, the distribution function shape parameters were estimated using an inverse FE approach by comparing strain distributions from the simulations with molecular strain obtained from XRD measurements under load (see Section 2.4.3). All numerical simulations showed good agreement with the experimental data. Additionally, the simulated strain distributions in the CT–LL and CT–PM transition regions (Figure 8e and Figure 9d, respectively) showed that in the absence of an explicit FGM model, numerical instabilities and unrealistic deformation characterized these regions. These results indicate that it is necessary to incorporate the graded properties in surgical simulators or whole-body FE models for injury prediction, such as those presented in this work.

**Figure 10:**
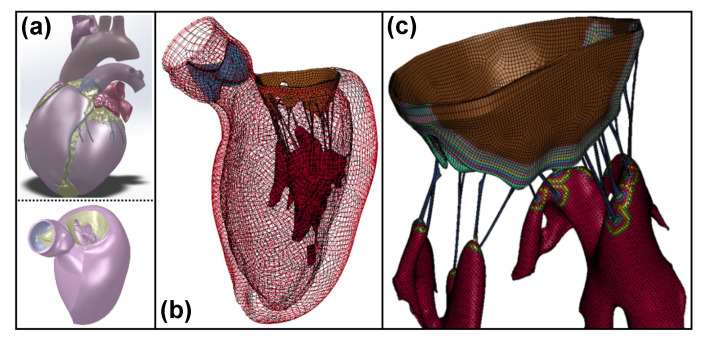
(**a**) Representative image of the whole heart and extracted left ventricle CAD (adopted from the Zygote Media Group, Inc. [58]), (**b**) meshed representation and (**c**) transition regions in the FGM model.

It must be emphasized here that our approach is different from structural constitutive models [20,24,28,32,57] as no new constitutive law is proposed to account for the spatial heterogeneity. A lumped representation using phenomenological models potentially reduces insights into the tissue substructure compared to structural models, nevertheless, the former is more amenable to numerical implementation [21], and the proposed FGM model is designed with the intent of preserving scalability and ease of backward integration in existing whole-body simulators that currently function without explicit transition modeling. A preliminary mesh generation and TI property assignment on a full system level model of the left ventricle of the human heart, developed from a commercial CAD utilizing human computed tomography and magnetic resonance imaging scan data (Zygote Solid 3D Human heart CAD [58]), is shown in Figure 10.

There are some limitations in our approach. First, the applicability of our approach is restricted to the tissue components and their transitions, and the associated scale at which the predominant deformation behavior in the tissue can be captured using the TI formulation from Weiss et al. [38]. For tissues such as the myocardium which exhibits strong orthotropy [16,17], or arterial walls with two families of primary fiber orientation [59], the proposed FGM model in its current form is not suitable, and standalone structural constitutive models might be necessitated for the tissue transition modeling.

Secondly, the uniaxial tension tests used to characterize the “pure” tissue specimens (PM, CT and LL) properties were along the primary fiber component, implying that the matrix and fiber responses could not be individually decomposed from the overall tension response. This could potentially lead to some inaccuracies in the fitted $C_{1}$ values in the these specimens. Use of biaxial studies could potentially isolate the matrix response, leading to higher accuracy in $C_{1}$ in these fits [60]. However, the matrix response is likely to be significantly lower than that of the fiber, which is also borne out from our characterization results (see Table 1), implying that the tension response is most likely dominated by the fiber stiffness terms ($C_{3}-C_{5}$, $\lambda^{*}$) since the specimens were loaded along their fiber orientation. This implies that any potential inaccuracy in the fitted $C_{1}$ parameter does not significantly impact the FGM model performance.

It should be mentioned here that due to the thinness of the specimens, (∼0.2 mm for LL and ∼0.9 mm for CT), it was not possible to measure fiber orientations and the molecular strains in the through-thickness direction, due to which the symmetry was assumed in the assignment of fiber orientation and collagen intensity data (through the corresponding distribution function) onto the FE mesh in the CT–LL and CT–PM transition regions. Since the XRD measurements are obtained on a volume-averaged basis, i.e., the XRD pattern is a cylindrical average of millions of collagen fibers through the thickness at the scanned location [61,62], we believe that the experimental results presented in Figure 8 and Figure 9 do represent an aggregated response in the through-fiber thickness direction in the CT–LL and CT–PM. Nevertheless, additional 3D XRD scanning data might further elucidate spatial variations in the through-thickness deformation characteristics.

It is also worth noting here that while we have assumed a monotonic variation of the constituent material properties in the transition region, certain tissue transitions, such as bone–tendon junctions display a non-monotonic stiffness variation [63]. While bone is typically modeled using elastic–plastic material models, a corresponding non-monotonic distribution function ϕ(t) can be readily incorporated in soft-tissue transitions if the underlying material composition data warrants such an implementation.

## 5. Conclusions and Future Work

Our eventual goal is to leverage the proposed FGM modeling work to develop a generalized biomimetic approach for attaching two morphologically similar yet biomechanically dissimilar materials using the CT–LL and CT–PM transition regions as a model biological system. We envision that the proposed FGM modeling approach can be extended to other skeletal soft-tissue transitions occurring within the human body.

Full characterization and validation of tissue transitions using the methods presented herein is recommended for improving the FGM model’s predictive abilities; however, qualitative improvement in the simulation kinematics can be produced even if the constituent distribution function and fiber orientation data are not available. For instance, repositioning simulations of articulated limbs consisting of hard transitions in bone–ligament–bone and bone–tendon–muscle complexes within whole-body injury biomechanics models such as the GHBMC [2] and CAVEMAN [3] can be made more biofidelic using the approaches outlined in this work.

## Figures and Tables

**Table 1:** Fitted TI parameters in porcine tricuspid valve (TV) and mitral valve (MV). Units of $C_{1}$–$C_{3}$ and $C_{5}$ in Pa. $C_{4}$ and $\lambda^{*}$ are dimensionless. A bulk modulus of *K* = 1.464 × 10^8^ Pa was assumed [39]. The parameter $C_{6}$ has not been shown since $C^{0}$ continuity has been assumed in Equation (5), however, $C^{1}$ continuity has not been assumed at $\tilde{\lambda }=\lambda^{*}$. Additionally shown are the $R^{2}$ for the fits.

**Porcine TV**							
	$C_{1}$	$C_{2}$	$C_{3}$	$C_{4}$	$C_{5}$	$\lambda^{*}$	$R^{2}$
CT	3.00 × 10${}^{7}$	0.0	0.70 × 10${}^{4}$	80.00	5.441 × 10${}^{8}$	1.086	0.996
LL	1.95 × 10${}^{4}$	0.0	1.00 × 10${}^{5}$	13.50	4.80 × 10${}^{7}$	1.28	0.995
PM	0.30 × 10${}^{4}$	0.0	0.50 × 10${}^{2}$	28.50	1.024 × 10${}^{5}$	1.15	0.814
**Porcine MV**							
	$C_{1}$	$C_{2}$	$C_{3}$	$C_{4}$	$C_{5}$	$\lambda^{*}$	$R^{2}$
CT	3.37 × 10${}^{6}$	0.0	8.82 × 10${}^{3}$	60.00	9.21 × 10${}^{7}$	1.086	0.991
LL	3.00 × 10${}^{5}$	0.0	9.00 × 10${}^{4}$	40.00	5.38 × 10${}^{6}$	1.010	0.956
PM	1.05 × 10${}^{4}$	0.0	0.50 × 10${}^{3}$	24.50	1.111 × 10${}^{5}$	1.09	0.996

## Abbreviations

The following abbreviations are used in this manuscript:

TI	Transverse isotropy/transversely isotropic
FGM	Functionally graded material
FE	Finite element
MV	Mitral valve
TV	Tricuspid valve
LL	Leaflet
PM	Papillary muscle
CT	Chordae tendinae
XRD	X-Ray diffraction
MTJ	Muscle–tendon junction
CAD	Computer-aided design

## References

[1] Olasky J., Sankaranarayanan G., Seymour N., Magee J., Enquobahrie A., Lin M., Aggarwal R., Brunt L., Schwaitzberg S., Cao C.G. (2015). Identifying opportunities for virtual reality simulation in surgical education: A review of the proceedings from the innovation, design, and emerging alliances in surgery (IDEAS) conference: VR surgery. Surg. Innov..

[2] Park G., Kim T., Panzer M., Crandall J. (2016). Validation of shoulder response of human body finite-element model (GHBMC) under whole body lateral impact condition. Ann. Biomed. Eng..

[3] Butz K., Spurlock C., Roy R., Bell W., Barrett P., Ward A., Xiao X., Shirley A., Welch C., Lister K. (2017). Development of the CAVEMAN human body model: Validation of lower extremity sub-injurious response to vertical accelerative loading. Stapp Car Crash J..

[4] Tidball J. (1991). 12 Myotendinous Junction Injury in Relation to Junction Structure and Molecular Composition. Exerc. Sport Sci. Rev..

[5] Sedransk K., Grande-Allen K., Vesely I. (2002). Failure mechanics of mitral valve chordae tendineae. J. Heart Valve Dis..

[6] Hedia H., Mahmoud N. (2004). Design optimization of functionally graded dental implant. Bio-Med. Mater. Eng..

[7] Leong K., Chua S., Sudarmadji N., Yeong W. (2008). Engineering functionally graded tissue engineering scaffolds. J. Mech. Behav. Biomed..

[8] Charvet B., Ruggiero F., Guellec D.L. (2012). The development of the myotendinous junction: A review. Muscles Ligaments Tendons J..

[9] Liao J., Vesely I. (2004). Relationship between collagen fibrils, glycosaminoglycans, and stress relaxation in mitral valve chordae tendineae. Ann. Biomed. Eng..

[10] Herring S., Grimm A., Grimm B. (1984). Regulation of sarcomere number in skeletal muscle: A comparison of hypotheses. Muscle Nerve.

[11] Avazmohammadi R., Soares J., Li D., Raut S., Gorman R., Sacks M. (2019). A contemporary look at biomechanical models of myocardium. Annu. Rev. Biomed. Eng..

[12] Prot V., Skallerud B., Holzapfel G. (2007). Transversely isotropic membrane shells with application to mitral valve mechanics. Constitutive modelling and finite element implementation. Int. J. Numer. Meth. Eng..

[13] Prot V., Skallerud B., Holzapfel G. (2010). On modelling and analysis of healthy and pathological human mitral valves: Two case studies. J. Mech. Behav. Biomed..

[14] Yin F., Strumpf R., Chew P., Zeger S. (1987). Quantification of the mechanical properties of noncontracting canine myocardium under simultaneous biaxial loading. J. Biomech..

[15] Sun W., Abad A., Sacks M. (2005). Simulated bioprosthetic heart valve deformation under quasi-static loading. J. Biomech. Eng..

[16] Costa K., Holmes J., McCulloch A. (2001). Modelling cardiac mechanical properties in three dimensions. Philos. Trans. R. Soc. A.

[17] Sun W., Sacks M. (2005). Finite element implementation of a generalized Fung-elastic constitutive model for planar soft tissues. Biomech. Model. Mechanobiol..

[18] Pope A., Sands G., Smaill B., LeGrice I. (2008). Three-dimensional transmural organization of perimysial collagen in the heart. Am. J. Physiol.-Heart C.

[19] Billiar K., Kristen L., Sacks M. (2000). Biaxial mechanical properties of the native and glutaraldehyde-treated aortic valve cusp: Part II—A structural constitutive model. J. Biomech. Eng..

[20] Freed A., Einstein D., Vesely I. (2005). Invariant formulation for dispersed transverse isotropy in aortic heart valves. Biomech. Model. Mechanobiol..

[21] Sacks M. (2000). Biaxial mechanical evaluation of planar biological materials. J. Elast..

[22] Toma M., Jensen M., Einstein D., Yoganathan A., Cochran R., Kunzelman K. (2016). Fluid–structure interaction analysis of papillary muscle forces using a comprehensive mitral valve model with 3D chordal structure. Ann. Biomed. Eng..

[23] Kunzelman K., Einstein D., Cochran R. (2007). Fluid–structure interaction models of the mitral valve: Function in normal and pathological states. Philos. Trans. R. Soc. B.

[24] Hunter P., Nash M., Sands G. (1997). Computational Electromechanics of the Heart. Computational Biology of the Heart.

[25] Holzapfel G., Ogden R. (2009). Constitutive modelling of passive myocardium: A structurally based framework for material characterization. Philos. Trans. R. Soc. A.

[26] Gasser C., Ogden R., Holzapfel G. (2006). Hyperelastic modelling of arterial layers with distributed collagen fibre orientations. J. R. Soc. Interface.

[27] Kroon M., Holzapfel G. (2008). A new constitutive model for multi-layered collagenous tissues. J. Biomech..

[28] Zhang W., Ayoub S., Liao J., Sacks M.S. (2016). A meso-scale layer-specific structural constitutive model of the mitral heart valve leaflets. Acta Biomater..

[29] Sun W., Martin C., Pham T. (2014). Computational modeling of cardiac valve function and intervention. Annu. Rev. Biomed. Eng..

[30] Sacks M., Sun W. (2003). Multiaxial mechanical behavior of biological materials. Annu. Rev. Biomed. Eng..

[31] Chen L., Yin F., May-Newman K. (2004). The structure and mechanical properties of the mitral valve leaflet-strut chordae transition zone. J. Biomech. Eng..

[32] Rego B., Sacks M. (2017). A functionally graded material model for the transmural stress distribution of the aortic valve leaflet. J. Biomech..

[33] Baillargeon B., Rebelo N., Fox D., Taylor R., Kuhl E. (2014). The living heart project: A robust and integrative simulator for human heart function. Eur. J. Mech. A Solid.

[34] Gao H., Feng L., Qi N., Berry C., Griffith B., Luo X. (2017). A coupled mitral valve—Left ventricle model with fluid–structure interaction. Med. Eng. Phys..

[35] Hayes A., Vavalle N., Moreno D., Stitzel J., Gayzik S. (2014). Validation of simulated chestband data in frontal and lateral loading using a human body finite element model. Traffic Inj. Prev..

[36] Newell N., Salzar R., Bull A., Masouros S. (2016). A validated numerical model of a lower limb surrogate to investigate injuries caused by under-vehicle explosions. J. Biomech..

[37] Schwartz D., Guleyupoglu B., Koya B., Stitzel J., Gayzik S. (2015). Development of a computationally efficient full human body finite element model. Traffic Inj. Prev..

[38] Weiss J., Maker B., Govindjee S. (1996). Finite element implementation of incompressible, transversely isotropic hyperelasticity. Comp. Method Appl. M..

[39] Pena E., Calvo B., Martinez M., Doblare M. (2006). A three-dimensional finite element analysis of the combined behavior of ligaments and menisci in the healthy human knee joint. J. Biomech..

[40] Weiss J. (1995). A Constitutive Model and Finite Element Representation for Transversely Isotropic Soft Tissues. Ph.D. Thesis.

[41] Shim V.B., Fernandez J.W., Gamage P.B., Regnery C., Smith D.W., Gardiner B.S., Lloyd D.G., Besier T. (2014). Subject-specific finite element analysis to characterize the influence of geometry and material properties in Achilles tendon rupture. J. Biomech..

[42] Hallquist J. LS-DYNA Theory Manual. http://www.lstc.com/pdf/ls-dyna_theory_manual_2006.pdf.

[43] Maas S., Ellis B., Ateshian G., Weiss J. (2012). FEBio: Finite elements for biomechanics. J. Biomech. Eng..

[44] Madhurapantula R., Krell G., Morfin B., Roy R., Lister K., Orgel J. (2020). Advanced methodology and preliminary measurements of molecular and mechanical properties of heart valves under dynamic strain. Int. J. Mol. Sci..

[45] Chi S., Chung Y. (2006). Mechanical behavior of functionally graded material plates under transverse load—Part I: Analysis. Int. J. Solids Struct..

[46] Anani Y., Rahimi G. (2015). Stress analysis of thick pressure vessel composed of functionally graded incompressible hyperelastic materials. Int. J. Mech. Sci..

[47] Madhurapantula R., Eidsmore A., Modrich C., Orgel J. (2017). New Methodology and Preliminary Data in the Characterization of the Muscle Tendon Junction of Mammalian Muscle Tissues. EMS Eng. Sci. J..

[48] Adams B., Bohnhoff W., Dalbey K., Eddy J., Eldred M., Gay D., Haskell K., Hough P., Swiler L. (2009). DAKOTA, A Multilevel Parallel Object-Oriented Framework for Design Optimization, Parameter Estimation, Uncertainty Quantification, and Sensitivity Analysis: Version 5.0 User’s Manual.

[49] Chi S., Chung Y. (2006). Mechanical behavior of functionally graded material plates under transverse load—Part II: Numerical results. Int. J. Solids Struct.

[50] Bianchi F., Hofmann F., Smith A., Thompson M. (2016). Probing multi-scale mechanical damage in connective tissues using X-ray diffraction. Acta Biomater..

[51] Lee C.H., Zhang W., Liao J., Carruthers C., Sacks J., Sacks M. (2015). On the presence of affine fibril and fiber kinematics in the mitral valve anterior leaflet. Biophys. J..

[52] Billiar K., Sacks M. (1997). A method to quantify the fiber kinematics of planar tissues under biaxial stretch. J. Biomech..

[53] Liao J., Yang L., Grashow J., Sacks M. (2007). The relation between collagen fibril kinematics and mechanical properties in the mitral valve anterior leaflet. J. Biomech. Eng..

[54] Varma S., Orgel J., Schieber J. (2016). Nanomechanics of type I collagen. Biophys. J..

[55] Fratzl P., Misof K., Zizak I., Rapp G., Amenitsch H., Bernstorff S. (1998). Fibrillar structure and mechanical properties of collagen. J. Struct. Biol..

[56] Neice R. (2019). Development and Validation of a Pelvis Finite Element Model for Side Panel Intrusion Threats. Master’s Thesis.

[57] Holzapfel G., Gasser T., Ogden R. (2000). A new constitutive framework for arterial wall mechanics and a comparative study of material models. J. Elast..

[58] Solid 3D Human Heart Model. https://www.zygote.com/cad-models/solid-3d-human-anatomy/solid-3d-human-heart.

[59] Holzapfel G., Ogden R. (2010). Constitutive modelling of arteries. Philos. Trans. R. Soc. A.

[60] Feng Y., Okamoto R., Genin G., Bayly P. (2016). On the accuracy and fitting of transversely isotropic material models. J. Mech. Behav. Biomed..

[61] Orgel J., Miller A., Irving T., Fischetti R., Hammersley A., Wess T. (2001). The in situ supermolecular structure of type I collagen. Structure.

[62] Orgel J., Irving T. (2014). Advances in Fiber Diffraction of Macromolecular Assembles. Encyclopedia of Analytical Chemistry: Applications, Theory and Instrumentation.

[63] Genin G., Kent A., Birman V., Wopenka B., Pasteris J., Marquez P., Thomopoulos S. (2009). Functional grading of mineral and collagen in the attachment of tendon to bone. Biophys. J..

